# Cardiovascular disease: everybody’s business

**DOI:** 10.1016/j.clinme.2026.100630

**Published:** 2026-07-21

**Authors:** Tevfik F. Ismail, Ponnusamy Saravanan

**Affiliations:** Guy’s and St Thomas’ Hospital, London, UK; King’s College London, London, UK; Warwick Medical School, Warwick Applied Health, University of Warwick, Coventry, UK; Centre for Global Health, University of Warwick, Coventry, UK; Centre for Early Life, University of Warwick, Coventry, UK; Diabetes, Endocrinology and Metabolism, George Eliot Hospital NHS Trust, Nuneaton, UK

The last 50 years have seen remarkable advancements in our understanding and care of people with cardiovascular disease, driving associated mortality down by over 75%.^1^ However, this decline has slowed in recent years and, as patients are living longer and increasingly surviving cardiovascular events, the prevalence of cardiovascular disease continues to rise. Cardiovascular or circulatory disorders account for nearly 1.2 million hospital admissions a year and also represent a common comorbidity among those admitted for other reasons.^1^ Every physician, regardless of their specialty, will therefore encounter cardiovascular disease in their day-to-day practice. As such, the management and prevention of cardiovascular disease has to be everybody’s business if we are to effectively rise to the challenge of dealing with the growth in cardiovascular morbidity. With this in mind, this month’s cardiology CME section assembles a series of review articles that cover a spectrum of topics that physicians are likely to encounter. My co-author, Dr Tevfik Ismail, has carefully commissioned topics that are relevant and timely for general physicians across the world, providing the latest updates on these areas. A brief summary of these articles is below:

Arguably no subspecialty within cardiology has seen advances in care as dramatic as those in heart failure over the last 40 years. For patients with reduced ejection fraction, we now have four effective drug classes of therapy, which have been shown in large randomised controlled trials to reduce mortality and/or heart failure hospitalisation.^2^ However, the care of patients with heart failure with preserved ejection fraction has historically been challenging and, until recently, was characterised by the absence of effective treatments and poor outcomes. This syndrome is now likely to be the dominant cause of heart failure and is associated with multimorbidity.^3^, ^4^ In this issue of the journal, Zakeri and Nabeebaccus outline recent advances in the care of people with this condition, give practical advice on diagnosis and referral and most importantly, outline new evidence-based treatment options which are finally beginning to bring hope of improved outcomes for these patients.^5^

One of the reasons for the stable or falling incidence of heart failure with reduced ejection fraction is improved prevention and management of ischaemic heart disease.^3^ The widespread use of statins for the primary prevention of atherosclerotic cardiovascular disease is in large part credited with falling cardiovascular mortality.^6^, ^7^ Blood pressure control, addressed in the May edition of the journal, also plays a vital role.^8^ Most people are able to take statins without any significant adverse effects and with the benefits far outweighing the risks.^9^ However, not all patients can tolerate statins and among those who do, many do not achieve contemporary treatment goals with statin therapy alone. In this issue, Han and colleagues describe the indications and evidence to support the use of non-statin lipid-lowering therapies.^10^ While many of the relevant drugs remain the preserve of specialist lipid clinics, some are readily available for prescription in primary care and non-specialist secondary care settings. However, as in many areas of medicine, low levels of clinician awareness remain a major obstacle to wider adoption. Han *et al*. address this by providing practical guidance on when to initiate treatment and the options available, as well as when to refer patients for specialist care.

In the field of cardiac arrhythmia, within the cardiology community there is rightly a strong preoccupation and focus on the management of atrial fibrillation and ventricular tachycardia, both of which have been covered in previous CME editions.^11^, ^12^ However, comparatively little attention is paid to ventricular ectopic or premature beats, which are extremely common in the general population. These are often benign arrhythmias that can be managed conservatively, however, they can sometimes be harbingers of more serious cardiovascular disease or be frequent enough to require treatment themselves. Yap *et al*. provide practical advice on diagnosis, ECG interpretation, when to refer and an outline of treatment options.^13^

Staying with the theme of common conditions that occasionally cause problems, one in four of the general population will have a patent foramen ovale (PFO). Right-to-left shunting across a PFO can potentially give rise to stroke and paradoxical thromboembolism, among other problems. Thankfully, only a small minority - those with the largest shunts or defects - are at significant risk of complications. However, given the high prevalence of PFOs, establishing their role as causal in disease and therefore recommending closure can be challenging. Most physicians encounter this problem in the setting of cryptogenic stroke. In this edition of the journal, Wilmshurst outlines the challenges involved, when to consider investigating for a PFO, how this can be done and addresses the often vexing question of when and whether to close a defect.^14^

Finally, echocardiography has long been an important clinical bedside tool for the evaluation of acutely unwell patients in emergency and critical care environments. Until recently, it has largely remained within the domain of cardiologists and requires very expensive hardware and the associated expertise in image acquisition and interpretation to leverage its management changing benefits. However, advances in ultrasound equipment design and miniaturisation have led to increased availability of cheaper and more accessible point of care ultrasound (POCUS) technologies. These, when combined with AI-assisted image acquisition and interpretation, have the potential to democratise the availability of diagnostic ultrasound (including basic echocardiography) and usher in a new era of ʻultrasound stethoscopy’.^15^ POCUS is already the standard of care for guiding the placement of central vascular lines and related critical care procedures, however, the technology is capable of far more than this. Aboumarie outlines the evolution of POCUS from a basic tool for ruling in/ruling out major pathologies to one used for complex haemodynamic assessment.^16^ It seems inevitable that future generations of physicians will need to be as comfortable and adept at using POCUS techniques as current generations are using stethoscopes for cardiopulmonary evaluation. This article provides a primer on what this technology can do and its applications.

The July issue also features eight original articles,^17^, ^18^, ^19^, ^20^, ^21^, ^22^, ^23^ four review articles,^24^, ^25^, ^26^, ^27^ two opinion pieces,^28^, ^29^ and an additional editorial on risk stratification of metabolic dysfunction-associated steatotic liver disease (MASLD).^30^ While I enjoyed reading every single one of them, here are my picks for this issue of *Clinical Medicine*. With increasing numbers of people living with obesity worldwide, the prevalence of MASLD is also increasing, along with other comorbid conditions associated with obesity. Although many guidelines, including NICE guidance, emphasise the importance of indentifying undiagnosed MASLD, a clear pathway for proactive identification and risk stratification of MASLD in primary care does not exist in most healthcare systems. Silva-Tinoco *et al*.^18^ tried to address this in a Mexican population and made a valuable addition to the existing evidence as highlighted by the accompanying editorial.^30^ Addressing this gap in evidence should be a priority rather than an optional extra for all healthcare systems. There is still a lot to learn on the prediction of MASLD, the pathophysiology of sub-types such as lean MASLD, which is more common in Asian and South Asian phenotypes, and the consequences of it, including cardiovascular disorders (CVD). Chauhan *et al*. review the unique aspects of dyslipidaemia and CVDs in South Asians and the need for dedicated, focused research with one of the commonest risk factors for CVDs.^24^ It is indeed true that common questions such as, a) should South Asians receive statins at the same thresholds of LDL or 10-year CVD risk score, or b) what is the efficacy of statins in CVD risk reduction etc have not been categorically answered. This review summarises the key differences compared to White ethnicity and calls for targeted research in some of the key aspects of dyslipidaemia. Safiri *et al*.^19^, in their analysis using the global burden of disease data on how the burden of rheumatic heart disease is still on the rise in many resource-constrained settings, highlight the importance of concerted efforts by the global medical community and policy makers in reducing avoidable mortality and morbidity. One of the short communications is also worthy of the attention of the medical fraternity and policy makers. While many policymakers across the world call for and in many cases are already implementing the ban of mobile phones in schools, it is our responsibility to highlight that such blanket ban could potentially do harm. One such situation is the use of ‘closed loop’ system (artificial pancreas) in type 1 diabetes which requires the continuous need for a mobile phone device with the children. Children with many other conditions also use mobile phone devices, and technological advances are improving care across a range of conditions. The authors therefore call for the labelling of these as medical devices and for more research in this area on the potential impact of any such blanket ban.^28^

Finally, two articles make a compelling case for how medical education can potentially help to address the challenges faced by the medical profession across the world. Arfeen *et al*.^26^ beautifully summarise the current key challenges for healthcare systems: globalisation, widening inequalities potentially worsened by rapid technological advantages and workforce shortages. In addition to providing historical context on colonial legacy and the impact on individual countries, they articulate how medical education can be a bridging force to mitigate these issues as well as help to reconcile the conflict between global geopolitical policies on immigration and the domestic needs of an individual country. Grant^25^ offers a different perspective on the ‘quality’ of postgraduate medical education. In the era of social media and increasing influence of AI on the provision of medical knowledge, the debate on ‘quality of care’ and ‘quality of education’ is more important than ever. This is in the context of differing styles of assimilation of medical knowledge by the current generation of resident doctors and their ability to adopt technological advances faster than senior medical professionals. I strongly recommend reading, digesting and reflecting on these articles!

## Funding

This article did not receive any specific grant from funding agencies in the public, commercial, or not-for-profit sectors.

## Declaration of competing interest

Dr Tevfik F. Ismail is the handling editor who commissioned the cardiology CME articles and deputy editor in chief, and Prof Saravanan is the editor in chief of Clinical Medicine.
